# Conceptual Development of Immunotherapeutic Approaches to Gastrointestinal Cancer

**DOI:** 10.3390/ijms20184624

**Published:** 2019-09-18

**Authors:** Bilikis Aderonke Abolarinwa, Ridwan Babatunde Ibrahim, Yen-Hua Huang

**Affiliations:** 1 International PhD Program for Cell Therapy and Regeneration Medicine, College of Medicine, Taipei Medical University, Taipei 11031, Taiwan; Bekee02@gmail.com; 2 Institute of Brain Science, School of Medicine, National Yang-Ming University, Taipei 11221, Taiwan; geniusridwan@gmail.com; 3 Taiwan International Graduate Program (TIGP) in Interdisciplinary Neuroscience, National Yang-Ming University and Academia Sinica, Taipei 11529, Taiwan; 4 Department of Biochemistry and Molecular Cell Biology, School of Medicine, College of Medicine, Taipei Medical University, Taipei 11031, Taiwan; 5 Graduate Institute of Medical Sciences, College of Medicine, Taipei Medical University, Taipei 11031, Taiwan; 6 TMU Research Center for Cell Therapy and Regeneration Medicine, Taipei Medical University, Taipei 11031, Taiwan; 7 Center for Reproductive Medicine, Taipei Medical University Hospital, Taipei 11031, Taiwan; 8 Comprehensive Cancer Center of Taipei Medical University, Taipei 11031, Taiwan; 9 TMU Research Center of Cancer Translational Medicine, Taipei Medical University, Taipei 11031, Taiwan; 10 Ph.D. Program for Translational Medicine, College of Medical Science and Technology, Taipei Medical University, Taipei 11031, Taiwan

**Keywords:** gastrointestinal cancer, predictive biomarker, immunotherapy, immune system, tumor microenvironment

## Abstract

Gastrointestinal (GI) cancer is one of the common causes of cancer-related death worldwide. Chemotherapy and/or immunotherapy are the current treatments, but some patients do not derive clinical benefits. Recently, studies from cancer molecular subtyping have revealed that tumor molecular biomarkers may predict the immunotherapeutic response of GI cancer patients. However, the therapeutic response of patients selected by the predictive biomarkers is suboptimal. The tumor immune-microenvironment apparently plays a key role in modulating these molecular-determinant predictive biomarkers. Therefore, an understanding of the development and recent advances in immunotherapeutic pharmacological intervention targeting tumor immune-microenvironments and their potential predictive biomarkers will be helpful to strengthen patient immunotherapeutic efficacy. The current review focuses on an understanding of how the host-microenvironment interactions and the predictive biomarkers can determine the efficacy of immune checkpoint inhibitors. The contribution of environmental pathogens and host immunity to GI cancer is summarized. A discussion regarding the clinical evidence of predictive biomarkers for clinical trial therapy design, current immunotherapeutic strategies, and the outcomes to GI cancer patients are highlighted. An understanding of the underlying mechanism can predict the immunotherapeutic efficacy and facilitate the future development of personalized therapeutic strategies targeting GI cancers.

## 1. Introduction

Gastrointestinal cancer (GI) is one of the deadliest malignancies, accounting for 3.3 million deaths worldwide according to the 2018 Globocan report. Patients are often diagnosed at a late and advanced stage and thus have limited treatment options. Approximately 20–25% of patients develop metastasis during the course of the disease [1]. Despite a meaningful prognosis and targeted chemotherapies, the overall survival rate in these patients still remains low [2]. Immunotherapeutic strategies have been successfully used in the treatment of melanoma, non-small cell lung cancer (NSCLC), and hematological malignancies [3,4]. However, these treatments have yielded an undesirable objective response rate (ORR) between 10% and 25% in GI cancers [5]. Understanding the tumor immune-microenvironment and potential predictive biomarkers will be helpful in strengthening patients’ immunotherapeutic responses [6].

This review focuses on the common GI cancers, specifically pancreatic cancer (PC), colorectal cancer (CRC), and hepatocellular carcinoma (HCC). Four points are discussed here: (1) The influence of pathogen-specific microbes and viral infections on GI cancers in light of chronic inflammation in pathogenesis. (2) The paracrine effect of fibroblasts and how a tumor modulates innate and adaptive immunity. (3) The emerging predictive biomarkers from molecular subtyping that contribute to the tumor immune landscape. (4) The existing and ongoing clinical trials on immunotherapy and their outcomes in GI cancers.

## 2. Crosstalk between the Immune System and Microbiomes on Homeostatic Regulation in GI Cancer

**Figure 1:**
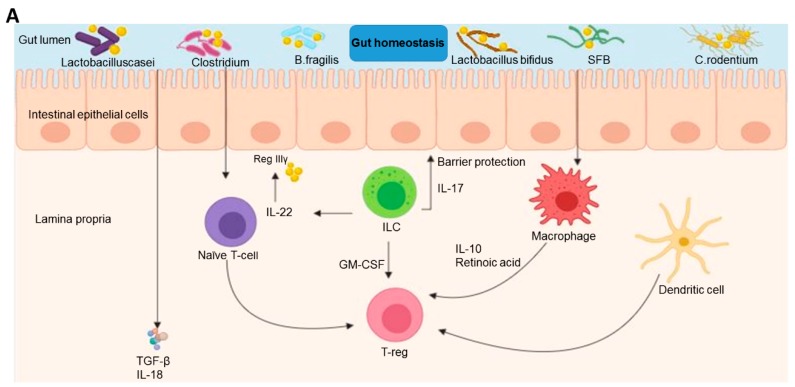

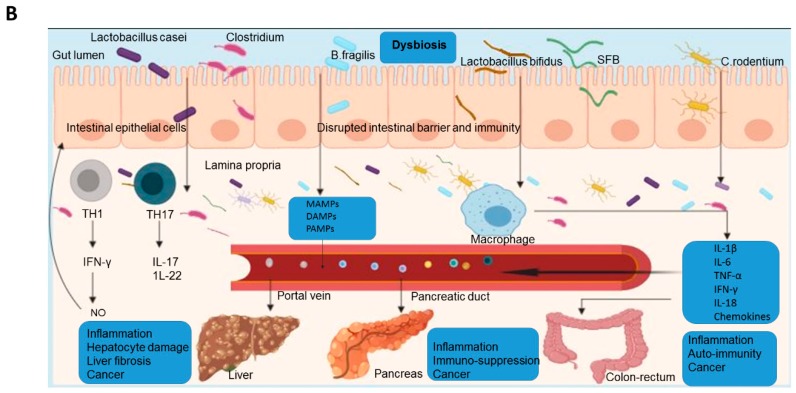
Intestinal homeostasis and dysbiosis of gut microbiomes in gastrointestinal cancer development. (**A**) Gut homeostasis. The gut homeostasis is maintained by an intricate network of factors such as regenerating islet-derived III-gamma (RegIIIγ), interleukin-22 (IL-22) and interleukin-17 (IL-17) secreted by innate lymphoid cells (ILC). These factors regulate the diverse gut microbiome, aid tissue repair, and barrier protection. Inhibition of immune activation by regulatory T cells (Treg) is aided by interleukin-10 (IL-10), retinoic acid and granulocyte macrophage-colony stimulating factor (GM-CSF) secreted by macrophage and innate lymphoid cells (ILC) respectively. In addition, secreted TGF-β and IL-18 preserve the intestinal barrier integrity and also promotes early development of regulatory T cells (Tregs). (**B**) Dysbiosis. On the other hand, an altered gut barrier due to a dysregulated microbiome disrupts the intestinal barrier resulting to leakage of microorganism associated molecular patterns (MAMPs), pathogens associated molecular patterns (PAMPs) and death associated molecular patterns (DAMPs) into the lamina propria. In addition, T helper-1 and T helper-17 produce interferon-gamma (IFN-γ), interleukin-17 (IL-17), interleukin -22 (IL-22) and excess nitric oxide, which induce loss of barrier integrity. Macrophages secretes inflammatory factors such as tumor necrosis factor (TNF-α), interferon- gamma (IFN-γ), interleukin-1 beta (IL-1β), interleukin -6 (IL-6), interleukin -16 (IL-16), interleukin-17 (IL-17), interleukin-18 (IL-18), and chemokines. These factors translocate via the portal vein and pancreatic duct to the liver and pancreas, respectively, and do so directly unto the colon–rectum, thereby initiating inflammation and cancer.

Gut microbiota are comprised of approximately 100 trillion diverse micro-organisms encompassing a varied taxonomy of 2000 distinct species. The microbes contain approximately 5,000,000 genes, which is 100–150 times higher than the genes in the human genome [6]. In healthy humans, the dominant bacterial phyla are Firmicutes (30–50%), Bacteroidetes (20–40%), and Actinobacteria (1–10%) [7]. Gut microbiota play a protective role against disease and a modulatory effect on immune cells [8]. They are also required to stimulate the proper development of gut-associated lymphoid tissues, including the Peyer’s patches, crypt, and other structures, while regulating helper T cell-mediated immunity [9]. Commensal bacteria colonize the host at birth and are essential to host development by priming the metabolic, immune, and nervous systems [10,11]. The mucosa of both the small and large intestine contain many scattered lymphocytes and lymphatic nodules, each of which are covered by columnar epithelial cells and mucus secreting goblet cells [12]. Homeostasis within the colon is largely maintained by the interaction between intestinal microbes and the immune system, mediated by intestinal epithelial cells. The relationships between microbiome dysbiosis and CRC have been discussed [13,14]. The interaction between the liver–pancreas and gut microbiota is indirect. In instances where a certain assault compromises the intestinal barrier, gut-derived bacteria produce lipopolysaccharides (LPS) and other metabolites, which translocate through the portal vein and pancreatic duct, respectively (see Figure 1) [15].

Though the pancreas was initially presumed to be a sterile organ and not usually exposed to microbiota, Pushalkar et al. [16] reported a 1000-fold increase of the intrapancreatic bacteria of human pancreatic ductal adenocarcinoma (PDAC) compared with normal pancreatic tissue. Similarly, several studies have identified a relationship between microbiota, inflammation, and PC [17]. In addition, Sun et al. [18] found that pancreatic β-cells express cathelicidin-related antimicrobial peptide under the influence of gut microbiota, thereby exerting an immunoregulatory effect. By contrast, the liver is constantly exposed to microbiota through the liver sinusoid [19]. The liver is a powerful local surveillance and tolerogenic system with a unique blood supply that plays a role in immunological defense and homeostasis.

The liver’s first line of defense is conferred by the resident Kuppfer cells (KCs), which enhance hepatic tolerance by stimulating anti-inflammatory cytokines in response to endotoxins while suppressing T cells through the activation of a toll-like receptor (TLR2) [20]. Exposure of the liver to microbes enhances activation of KC via TLR3, which inhibits immune tolerance and induces T cell response [21]. In addition, liver sinusoid epithelial cells (LSEC) express most of the TLRs and present antigens directly to T cells [22]. Myeloid-derived suppressor cells (MDSCs) are potent activators of suppressive cytokines such as interleukin (IL)-10, IL-17, and transforming growth factor-beta (TGF-β), which downregulate the activity of both CD4^+^ and CD8^+^ T cells [23,24]. Natural killer (NK) cells within the liver respond to cell surface antigens caused by pathogens and stimulate both innate and adaptive responses through their ability to secrete various cytokines (e.g., IL-15, 1L-7, 1L-12, and interferon-gamma (IFN-γ) [25]. These cytokines are counterbalanced by TGF-β, IL-10, and IL-13 [26]. Hepatic NK cells also exert an immunomodulatory effect via pro-inflammatory and anti-inflammatory cytokines [25]. Therefore, any deregulation of the abovementioned intricate network of immune cells, gut microbiota, and intestinal barrier results in gastrointestinal diseases [27] (see Figure 1).

## 3. Influence of Pathogens and Role of Immune Cells in GI Cancer

### 3.1. Influence of Microbiomes on GI Cancer

**Figure 2:**
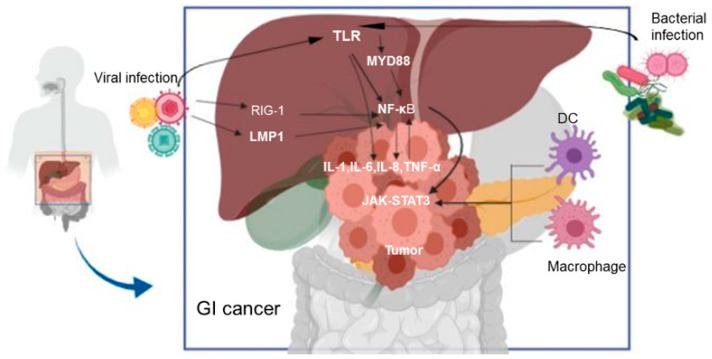
The mechanisms by which pathogens induce gastrointestinal cancer. Nuclear factor-kappa B (NF-κB) is stimulated through virus-induced activation of toll like receptor (TLR), retinoic acid-inducible gene-1 (RIG-1) and Epstein–Barr virus latent membrane protein 1 (LMP1). Bacterial infection also can activate TLR and myeloid differentiation primary response 88 (MYD88) to stimulate NF-κB, which in turn promotes pro-inflammatory cytokines; IL-6, IL-1β, IL-8, tumor necrosis factor-α (TNF-α) and vice versa. The activation of pro-inflammatory cytokines promotes infiltration of dendritic cell, macrophages and other immune cells which activates Janus kinase/signal transducer and activator of transcription 3 (JAK-STAT3). The inflammatory responses and NF-κB activation promotes cell proliferation and cancer initiation. In addition, the cross-talk between (NF-κB) and JAK-STAT3 stimulate cell growth, angiogenesis and thus accelerate tumorigenesis.

The pro-tumorigenic role of gut dysbiosis has been described in different cancers through multiple mechanisms, including metabolic changes, inflammatory cytokines, oncogenic pathways, and adhesion molecules [28]. The bacteria and its metabolites contributes to gastrointestinal cancers. *Porphyromonas gingivalis*, an important contributor for systemic inflammation, was shown in higher levels in PC patients [29,30]. *P. gingivalis* is an activator of TLR which acts through the immunoglobulin (Ig)–like molecule (B7-H1) receptor and its mediated co-stimulatory signal. This promote the apoptosis of activated T cells [31,32]. Similarly, the proteobacteria (gut microbiota) within the tumor microenvironment have been shown to promote immune suppression through the activation of toll-like receptors in monocytic cells [16]. Hence, proteobacteria ablation results in the immunogenic reprogramming of the tumor microenvironment through enhanced T helper-1 (TH1) differentiation of CD4^+^ and up-regulation of programmed cell death- 1(PD-1) expression [16]. Additionally, the liver tissue is the most common metastatic organ for PC. The recruitment of granulin-secreting inflammatory monocytes to the liver reprograms hepatic stellate cells into myofibroblasts, which supports the growth of metastasizing tumor cells [33]. The accumulation of lipopolysaccharides contributes to the pathogenesis of HCC by activating pro-inflammatory cytokines through toll-like receptor 4 (TLR-4) [34]. TLR activates innate immunity through myeloid differentiation primary-response protein 88-dependent (MyD88) and MyD88-independent pathways [35] (see Figure 2).

Mice deficient in both TLR-4 and MyD88 have shown a significant decrease in the incidence and sizes of chemical-induced liver cancers, suggesting a strong relationship between TLR-4 signaling and hepatocarcinogenesis [36]. Several bacteria such as *Fusobacterium nucleatum*, *Escherichia coli*, *Bacteroides fragilis*, and *Enterococcus faecalis* are elevated in CRC patients [37]. By contrast, *Clostridiales*, *Faecalibacterium*, *Blautia obeum*, and *Bifidobacterium* are absent within CRC [38]. Bacteria that colonize the surfaces of the caecum and colon induce inflammation through the T helper-1 and T helper-17 (Th1/Th17) immune response. This aids the recruitment of tumor-infiltrating myeloid cells and cancer progression [39,40]. Studies have shown that STAT3 (signal transducer and activator of transcription 3) activation contributes to inflammatory bowel disease and CRC [41,42]. Bacteria also activates ERK (extracellular signal-regulated kinase) and C-MYC, as demonstrated in an APC ^min/+^/MyD88^−/−^ mouse models [43]. Dejea et al. reported that 89% of right-sided and 12% of left-sided human CRC contain microbial biofilm [44]. Similarly, microbial biofilm from a healthy individual may be a point of transition from a healthy state to a diseased state [45]. Tomkovich et al. [46] demonstrated that microbial biofilm from CRC patients and healthy individuals induces tumor formation when transferred to germ-free mice. Additionally, the microbial biofilm from a CRC patient aggressively promoted tumor growth within one week compared with biofilm-positive homogenates from a healthy individual. Furthermore, the carcinogenic phenotype maintained in a new host is same as the phenotype from the biofilm source. Immune cells such as natural killer T (NKT) cells, myeloid cells, and Th17 were recruited by the biofilm in the germ-free mice. A contrasting role has been reported for Th17, given its involvement in biofilm-induced tumor formation. For example, it is pro-inflammatory through its enhanced secretion of IL-22 and IL-17 [47]. Conversely, an inflammatory-independent role has been reported in *F. nucleatum*-fed mice [39].

### 3.2. Influence of Virali Infection in GI Cancers

**Figure 3:**
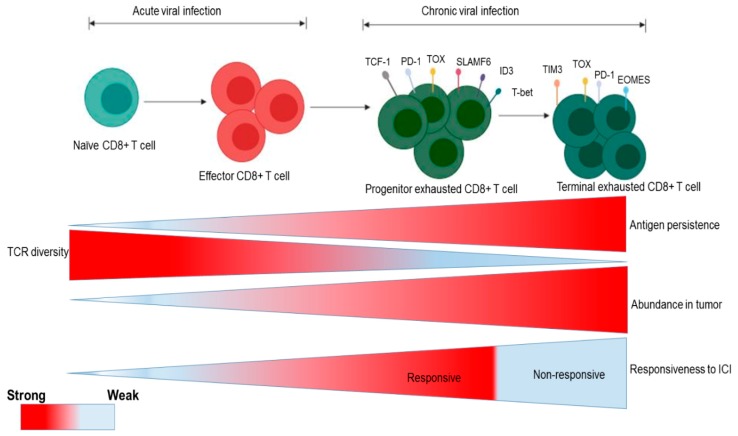
Viral infection promotes T cell exhaustion. The chronicity of viral infection determines the fate of CD8^+^ T cell. During acute viral infection, naïve CD8^+^ T cells differentiate into effector CD8^+^ T cells. These subset of effector CD8^+^ T cells possess an effective cytotoxic activity through diverse T cell receptor (TCR). However, chronic viral infection promotes antigen persistence, causing expression of exhaustion markers on T cells and a phenotype switch to an exhausted state. Exhausted T cells have two subpopulations; progenitor exhausted and terminally exhausted T cells. The progenitor exhausted T cells are responsive to immune checkpoint inhibitor (ICI), whereas terminally exhausted T cells are more abundant within the tumor and are non-responsive to immune checkpoint inhibitor (ICI).

Viral infection agents are risk factors of cancers. The role of human papilloma virus, hepatitis virus (B and C), coxsackie virus, cytomegalovirus (CMV), human immunodeficiency virus, herpes simplex virus, mumps, and varicella-zoster virus have been documented [48]. For example, chronic hepatitis B and C viruses are the leading cause of HCC worldwide [49]. These viruses are also present in the extrahepatic tissue contributing to extrahepatic metastasis in PC [50]. Similarly, transfusion-transmitted virus (TTV), one of the causative agents of hepatitis, has been detected in pancreatic tumor patients [51]. However, the nature of the link between PC and TTV is unknown. Till date, studies regarding the role of viral infection in PC is limited. The presence of viral DNA has been reported in colorectal tumor tissues. For instance, human papillomavirus has been detected in 1,549 samples [52] and BK virus has been detected in 50 clinical specimens [53]. More recently, Mjelle et al. identified micro RNA (miRNAs) from Epstein-Barr virus in CRC tumor samples [54]. Whether viral infections contribute to the development of CRC remains controversial. It is well documented that chronic inflammation is the cause of cancers [55]. Viral infections have been shown to directly initiate cell proliferation and promote inflammation [56]. Residues from death-associated molecular patterns (DAMP) and inflammasomes promote inflammatory cytokines, leading to HCC progression [57]. The inflammatory responses promote lymphocyte infiltration, macrophages, natural killer cells, dendritic cells, and pro-inflammatory cytokines (IL-6 and TNF), which activate STAT3 and NF-κB (nuclear factor-kappa B) [58]. NF-κB stimulates pro-inflammatory cytokines such as IL-6, IL-1β, IL-8, TNF-α and chemokines C-X-C motif ligand 1 and 2 (CXCL1 and CXCL2) [59] (see Figure 2). Additionally, pro-inflammatory cytokines exert a stimulatory effect on cyclo-oxygenase (COX)-2, which regulate immunity and maintain gastrointestinal integrity [60]. Hence, the inhibition of STAT3 and NF-κB abolish inflammation in animal models of HCC [61], PC [62], and CRC [63]. Conversely, the inhibition of NF-κB enhances HCC, thereby damaging hepatocytes in animal models of hepatitis B-virus (HBV)-driven HCC [64]. Therefore, NF-κB poses a challenge due to its opposing role in HCC. Furthermore, the persistent activation of the immune system due to viral infection results in organ damage due to a weakened and overpowered immune system [65], leading to T and NK cell exhaustion [66]. It has been shown that a memory-like virus-specific T cell is elevated without cessation despite the withdrawal of chronic antigen stimulation during viral infection [67]. Exhausted T cells express multiple inhibitory receptors such PD-1, lymphocyte activation gene-3 (LAG-3), T cell immunoreceptor with Ig ITIM domains (TIGIT) [68]. Similarly, exhausted NK cells also express NKG2A as a checkpoint molecule during viral infection. A recent study identified thymocyte selection-associated high mobility group box protein (TOX) as the main regulatory protein in exhausted T cells [69]. The similarities between virus-induced T cell exhaustion and dysfunctional T cells in cancer have been a major debate. However, limited knowledge exists on the similarities between dysfunctional T cells in cancer and exhausted T cells in viral infection. The persistent activation of CD8^+^ T cells results in loss of their effector function. This leads to dysfunctional T cells with molecular profiles that distinguishes them from exhausted cells in chronic viral infection [70]. By contrast, Miller et al. [71] identified a shared epigenetic program of exhaustion that is independent of the disease-specific milieu by comparing exhausted CD8^+^ T cells from mice infected with lymphocytic choriomeningitis virus and CD8^+^ T cells isolated from ovalbumin-expressing B16F10 (B16-OVA) mouse melanoma tumors. Studies from transcriptome analysis have shown high similarities between T cell dysfunction in HCC and exhausted T cell from chronic HBV infection. However, viral induced exhausted T cells and non-viral dysfunctional T cells possesses specific genes and signaling pathways [72]. Exhausted CD8^+^ T cells still have effector functions in both chronic viral infection-induced tumors and cancers without viral infection [71,73]. The effector function is regulated by a balance between the two major subpopulations of the exhausted T cells (progenitor exhausted and terminal exhausted cells) (see Figure 3).

The terminally exhausted T cells with a shorter life span have CD8^+^ T cell cytotoxic effector function [74]. By contrast, progenitor-exhausted cells have a longer life span but a poor cytotoxic effect [71].

### 3.3. Paracrine Effect of Fibroblasts on GI Cancer

Fibroblasts are spindle-shaped cells found within loose connective tissue [75]. They consist of protein fibers composed of collagen (collagenous fibers) scattered loosely within the confines of the extracellular matrix. Fibroblasts are activated not only in wound healing but also in cancer, and are referred to as cancer-associated fibroblasts (CAFs). CAFs are a major subset in the tumor microenvironment that provide cues to cancer cells in the form of secretions, aiding their growth in a paracrine manner [76]. Though the specific origin of CAFs remains controversial, studies have proposed that they consist of a diverse subset that is differentiated from a specific origin such as conventional fibroblasts in the early stages of a tumor [75], recruitment from bone marrow-derived mesenchymal stem cells [77], or non-fibroblast lineage (epithelial cell, blood vessels or serosa, and stem cell origin) [78]. More recently, a distinct population of CAFs was found in breast cancer and NSCLC. These populations express complement 5a G-protein coupled receptor-77 (GPR77) and CD10 with the ability to promotes stemness and chemoresistance through the NF-κB activation and cytokine secretion (IL-6 and IL-8) [79]. Furthermore, CAF have specific markers that are shared with other cell types. For instance, a fibroblast marker known as alpha smooth muscle (α-SMA) is also expressed by the smooth muscle cell of GIT. Ozdemir et al. [80] found that depleted α-SMA^+^ cells led to tumor progression in a PDAC mouse model. Conversely, the high expression of fibroblast activation protein (FAP) has been found to correlate with poor prognosis in both CRC and PDAC. Therefore, the depletion of FAP leads to impaired tumors in both types of cancer [81,82,83]. It is known that sonic hedgehog signaling pathways contribute to the development of tumors. However, the use of hedgehog inhibitors in pre-clinical and clinical studies has failed to alleviate tumor progression in both PDAC and CRC [84,85]. FAP^+^ CAFs modulate the tumor microenvironment through secretions such as C-C motif chemokine ligand 1, 2 and 5 (CCL1, CCL2, CCL5) and CXCL12 in HCC [86]. In addition, CAFs recruits monocytic MDSCs (M-MDSCs), regulatory T cells (Treg CD4^+^ CD25^+^ cells) and initiates macrophage polarization [87,88]. However, evidence has suggested that MDSCs recruited by CAF exert a neutralizing effect within the tumor microenvironment [89]. Inflammatory cytokines such as IL-6, IL-1, and TGF-β secreted by CAF inhibit both IFNγ secretion in PC and the tumoral infiltration of CD8^+^ T cells in colon cancer [90,91]. Recently, a study showed that both hepatocyte growth factor (HGF) and IL-6 secreted by CAF promote stemness in CD24^+^ HCC through STAT3 activation [92]. Colony stimulating factors (CSF) have been shown to modulate the crosstalk between CAFs and cancer cells via CXCL1 [89]. Clinical studies reported a minimal effect of the CSF-1 inhibitor on the disruption of such crosstalk in gastrointestinal tumors [93]. CAFs and CD90^+^ colonic (myo)fibroblasts express PD-1, which suppresses Th1 helper T cells in ulcerative-induced colitis patients [94] as well as CD8^+^ T cells [95]. In addition, CAF also exerts a modulatory effect on neutrophils by increasing PD-L1, IL-8, TNF-α, and CCL2 expression thereby inhibiting T cell response [96].

### 3.4. Drivers of Innate Immunity and GI Cancer

#### 3.4.1. NK Cells

NK cells are a component of innate immunity and the first line of defense against foreign agents within the body. They kill target cells without prior notice at first sight and recruit the adaptive immune components to reinforce the immune response through cytokine secretion [97,98]. NK cells can be distinguished by the expression of CD16 and CD56, constituting CD56 bright/CD16^−^ (tissue NK cells) and CD56 dim/CD16^+^ (blood) subsets. The cytotoxic effect of NK cells are primarily attributed to the CD56 dim/CD16^+^ subset, which constitutes approximately 90% of NK cells [99]. CD 16 plays an important role in the activation of NK cells by binding to the Fragment crystallizable (Fc) portion of immunoglobulins and causing the release of cytokine, thereby recruiting adaptive immunity through antibody-dependent cell-mediated cytotoxicity [100]. The number of NK cells has been reported to outnumber CD8^+^ T cells in liver tumors [101]. Despite this, NK cell function is diminished in HCC. Several explanations for this phenomenon have been proposed, including NK cell exhaustion or dysfunction [102], low levels of liver-resident NK cells causing hypo functionality, non-resident cells maintaining much higher expression [103] and the upregulation TFG-β and IL-10 through the activation of STAT3 [104]. Similarly, a reduced level and activity of NK cells has been reported in patients with PC and CRC compared with healthy controls [105,106]. Another study showed that the activity of NK cell-induced interferon-γ is impaired in post-operative CRC patients, resulting in the recurrence and formation of early micro metastases [98]. Post-operative impaired NK cell interferon-γ secretion has been reported as a T cell suppressor in a study using OVA-specific T cells [107]. The impaired activity of NK cell and reduced level of NKG2D was reported in advanced PC. By contrast, increased NKG2D expression was reported in resected PC [108]. However, a recent study found no correlation between NKG2D and interferon-γ secreting NK cells [109].

#### 3.4.2. Dendritic Cells

Dendritic cells (DCs) are antigen-presenting cells that play a crucial role by inducing anti-tumor immune responses. The priming of the effector CD8^+^ T cell response by DCs has been well documented in several cancers [110]. Furthermore, a recent study demonstrated that IL-33 enhances and promotes the cytotoxic activities of a DC-induced CD8^+^ T cell newly identified subset 9 (Tc9). These activities inhibit the differentiation of exhaustive CD8^+^ T cells by decreasing expression of both 2B4 (CD244) and PD-1 while increasing IL-2 and CD127 (IL-7Rα receptor) expression on CD8^+^ T cells in an OT-I melanoma mouse model [111]. Despite the reported anti-tumor effect of dendritic cells, several studies have also shown their immunosuppressive role in human tumors including GI cancers. In HCC, FcγRII ^low/−^activated B cells are generated by semi-mature dendritic cells through CD95L-dependent pathway. The activated FcγRII ^low/−^ B cells from the HCC tumor subsequently suppress autologous tumor-specific cytotoxic T cell immunity through IL-10 [112]. Similarly, Yuan et al. [113] demonstrated an increased number of immature dendritic cells and a decreased number of mature dendritic cells in CRC tumor tissues. The decreased number of mature dendritic cells is associated with tumor escape from the immune system [114]. Furthermore, trefoil factor 2 was reported as the main chemoattractant for immature dendritic cells with no impact on its phenotypic maturation in pancreatic cancer [115]. Interestingly, a distinct subset of dendritic cells, CD103^–^CD11b^+^ DC, drives CD4^+^ T cell tolerance and also express TGF-β, IL-23 and IL-10^+^ IL-17^+^ FOXP3^neg^ Tregs (Tr1 cells) [116]. The ablation of the CD103^–^CD11b^+^ DC subset mitigates the CD4^+^ T cell expression of IL-17 and retinoic acid receptor-related orphan receptor gamma-t (RORγt) in PDA-bearing CD11c.DTR bone marrow chimeric mice [116]. Tr1 cells eliminate CD8^+^ T cells through perforin-granzyme B and also suppress innate immunity through IL-10 in myeloid cells [117,118]. The use of DCs in both HCC and CRCs as a therapeutic window is an ongoing area of research [119].

#### 3.4.3. Macrophages

Macrophages are cells of the mononuclear phagocyte system. There are two types of macrophage; namely M1 (activated) and M2 (alternatively activated), which expresses CD163. M1 is pro-inflammatory and anti-tumor, whereas M2 is anti-inflammatory, pro-tumorigenic, promotes angiogenesis and repairs damaged tissue [120]. Tumor associated macrophage (TAMs) are immune-related stromal cells which provide support for cancer cells [121] and have the ability to modulate chemoresistance by activating autophagy [122]. Studies have reported that recurrence and cancer-related death occur in patients with high TAM infiltration within the tumor stroma [123]. Consistently, evidence from animal studies has also shown a detrimental effect of high infiltrated TAM2 rather than the total TAM population [124]. The prevalence of high tumor-infiltrating TAM2 correlates with larger tumor size and poor prognosis in solid tumors, including PC [125] and HCC [126]. Studies have shown that TAM1 is localized to the perivascular niche, in contrast to the TAM2 which is found in hypoxic areas [127]. The C–C chemokine ligand type 2 and receptor (CCL2/CCR2) pathway is required for the mobilization of monocytes from the bone marrow into the tumor microenvironment [128]. Bartneck and colleagues [129] reported an accumulation of a distinct subset of macrophage, CCR2^+^ TAM at the stroma/tumor interface within a highly vascularized region in resected HCC. This subset of macrophage expresses an inflammatory marker S100a9 rather than CD163. Hence, the depletion of CCL2 results in reduced TAM, pathogenic angiogenesis and tumor progression. Additionally, IL-34, IL-35, IL-10, IL-4, TGF-β, and CSF-1 promote the differentiation, survival, and recruitment of macrophages into the tumor microenvironment [130,131,132,133]. The abrogation of colony-stimulating factor 1 and its receptor (CSF-1/CSF-1R) depletes CD206^hi^ TAMs and also reprograms residual TAMs to promote antigen presentation and T cells in an experimental model of PDAC [134]. As opposed to the beneficial effect of the CSF blockade, they also upregulate immune checkpoint molecules; PD-1 and cytotoxic T-lymphocyte-associated protein4 (CTLA-4). Another study also showed that TAMs can be derived from tumor-infiltrating monocytes, which express an elevated level of programmed cell death-ligand 1 (PD-L1), suppressing cytotoxic T cell responses [135]. Hence, the combination of a CSF inhibitor and an immune checkpoint antagonist inhibits tumor progression [134]. Consistently, the depletion of a specific extratumoral macrophage Ly6C^low^F4/80^+^ enhances CD8^+^ T cell tumor infiltration in response to CD40 agonist immunotherapy [136]. A study identified Kupffer cells as a TAM population that promotes tumor progression in HCC [137]. However, it is difficult to determine the functional role of KC due to a lack of animal experimental models that can selectively inhibit KC without resulting in liver toxicity to confirm the observation [138]. There is a contrasting evidence regarding the exact role of TAM in CRC, as it has been shown to exert both beneficial and detrimental effects on this type of cancer [139]. Khorana et al. [140] reported that vascular endothelial growth factor (VEGF)-expressing TAM increases median survival in patients with colon carcinoma, and a decreased number of macrophages is associated with more advanced stages among CRC patients. By contrast, pancreatic macrophages express VEGF-A, VEGF-C, and basic fibroblast growth factor (FGF), promoting angiogenesis and tumor invasiveness [141]. Of particular clinical relevance was a finding that macrophage orchestrates resistance to anti-VEGF therapy and macrophage depletion could improve VEGF blockade [142].

#### 3.4.4. Myeloid-Derived Suppressor Cells

**Figure 4:**
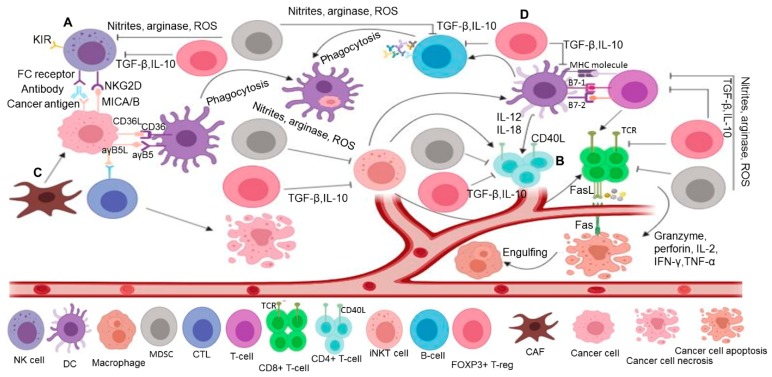
The role of immune cells in cancer development. (**A**) NK cells are the first line of innate immunity, activated by NKG2D and CD16 (Fc receptor) to promotes cancer cell killing through an antibody dependent cell-mediated cytotoxicity (ADCC effect). (**B**) The activation of CD4^+^ T cells, CD8^+^ T cells and B cells promotes phagocytosis of cancer cells by dendritic cells and macrophages through Fas/FasL, granzymes, perforins, interleukin-2 (IL-2), tumor necrosis factor (TNF-α), and interferon –γ (IFN-γ). (**C**) Cancer associated fibroblast (CAF) provides support for cancer cells to promotes immune escape. (**D**) By contrast, these anti-tumor function is counteracted by the presence of fork head box P3 (FOXP3^+^) T regs (regulatory T cell) and myeloid-derived suppressor cells (MDSC) through production of TGF-β, IL-10, arginase, nitrites and ROS.

Myeloid derived suppressor cells (MDSCs) are immature myeloid-derived cells. These cells suppress immune response through an array of secretory factors such as arginase, nitrites, reactive oxygen species (ROS), immunosuppressive cytokines and the expansion of immunosuppressive cells (Tregs) [143,144] (see Figure 4). Impaired myelopoiesis results in defective differentiated progenitor cells in cancer [145]. This alters their phenotype, similar to TAM and tumor-associated neutrophil, and these are referred to as M-MDSCs and polymorphonuclic or granulocytic (G-MDSC/PMN-MDSC) [146,147]. Murine MDSCs are characterized by the co-expression of CD11b, an α-M integrin, and the myeloid differentiation antigen Gr1 [148], whereas human M-MDSCs are characterized by CD11b^+^CD14^+^HLA-DR^low^/^−^CD15^−^ and PMN-MDSCs as CD14–CD11b^+^ CD15^+^ (or CD66b^+^) cells [149]. CD38, a transmembrane receptor-ectoenzyme, is highly expressed in both the M-MDSCs and G-MDSC/PMN-MDSC of CRC patients [150]. Studies have shown that the high infiltration of MDSC correlates with a poor prognosis in most cancers [151,152]. Consistently, reports from a KrasLSL.^G12D/+^; p53^R172H/+^; Pdx^Cretg/+^ (KPC) model of metastatic PC showed that MDSCs positively correlate with cancer cell metastases and suppresses T cell proliferation through GM-CSF [143]. Another study showed that a population of CD14^+^ HLA^−^DR^low^ M-MDSCs catabolizes L-arginine, which inhibits T cell-induced IFN-γ and NKp30-induced cytotoxicity in HCC [153,154]. However, the administration of exogenous L-arginine to the co-culture medium reversed IFN-γ secretion. Similarly, the accumulation of CD11b^+^Gr1^+^ MDSCs-induced IL-10 has also been shown to promote colitis-induced CRC through epigenetic upregulation of DNA methyl transferase (DNMT3b and DNMT1), as well as a decreased expression of interferon regulatory factor 8 [155].

### 3.5. Drivers of Adaptive Immunity and GI Cancer

#### 3.5.1. T Lymphocytes

The subset of total CD3^+^ T cells includes cytotoxic CD8^+^ T cells, CD4^+^ helper T cells and CD4^+^CD25^+^ Tregs. Cytotoxic CD8^+^ T cells physically attack foreign invaders through perforins, granzymes, and a FasL/receptor [156] (see Figure 4). By contrast, B lymphocytes kill at a distance through humoral immunity (the secretion of antibodies). Cytotoxic T cells secrete IFN-γ and TNF-α, and combat invading molecules through major histocompatibity complex (MHC) class I [157]. Helper T lymphocytes aid B lymphocytes and killer T lymphocytes through the secretion of chemical regulators called lymphokines (i.e., IL-22). Fork head box P3 (FOXP3)^+^ Tregs secrete IL-10 and TGF-β, which inhibit both B lymphocytes and T lymphocytes. CD8^+^ T cells are the main constituent of tumor-infiltrating lymphocyte (TILs) that perform the effector function. Studies have shown that the distribution and density of TILs determine the functional states within the tumor microenvironment (i.e., anti-tumorigenic or pro-tumorigenic) [158]. The distribution of TILs varies significantly among different cancer types. For instance, TILs are confined to the peritumoral tissue in PC patients [159] and both peritumoral and intra-tumoral in CRC patients [160]. A higher infiltration correlates with longer survival in human cancer [161,162]. The anti-tumor effector function requires the infiltration of CD8^+^ T cells within the tumor. Hence, a lower number or absence of CD8^+^ T cells within tumors have been implicated as major obstacles for immunotherapies in solid tumors, especially in PC [163]. Mounting evidence over the years has shown the potential mechanism responsible for the reduced TIL infiltration. Zhang et al. [164] reported that CD11b^hi^F4/80^low^ tumor-associated myeloid cells secrete the S100a9 protein through CCL5, which inhibits the accumulation of CD8^+^ T cells. The blockage of CCL5-enhanced CD8^+^ T cell mobilization and reduced secretion of the S100a9 protein in a CRC mouse model. In addition, the downregulation of type I interferon receptor (IFNAR1), which maintains the pool of CTL (cytotoxic T cell), promotes immune privileged niche [165]. The stabilization of IFNAR1 improves CTL and enhance the efficacy of chimeric antigen receptor T and PD-1 inhibition. Furthermore, the lack of neoantigens expressed in cancer cells may also result in poor TIL infiltration [166]. In addition, exosome derived 14-3-3ζ (14-3-3 protein zeta) results in an impaired anti-tumor effect of TILs, thereby contributing to T cell exhaustion [167]. The depletion of CD8^+^ T cells has been reported to promote tumor development in fumarylacetoacetate-induced hepatitis in fumarylacetoacetate hydrolase-deficient mice [168]. However, a study showed that the pro-tumorigenic role of CD 8^+^ T cells is due to lymphotoxin a and b, which promote chronic inflammation-induced tissue damage and HCC [169,170]. As opposed to their pro-tumorigenic role, lymphotoxins exert an anti-tumor response in numerous human cancers [171]. However, the mechanism underlying the switch from an anti-tumoral to a pro-tumorigenic effect is unknown. In addition, exosome-derived 14-3-3ζ (14-3-3 protein zeta) results in an impaired anti-tumor effect of TILs, thereby contributing to T cell exhaustion [167]. Studies have shown that tumor-specific CD4^+^ T cells have complex roles beyond supporting CD8^+^ T cells. CD4^+^ T cells are predominantly subtyped into Th1- and Th2-based on cytokine secretion. Th2 exerts an anti-tumor function, and an imbalance between Th1 and Th2 contributes to tumor progression. Studies have demonstrated an association between Th2 dominance and cancer [172], whereas Th1 confers a good prognosis in cancer [173]. Interestingly, CD4^+^ T cells have the ability to convert and switch to CD4^+^CD25^+^Treg [174]. Studies have shown that thymus-derived Tregs or peripheral-derived Tregs may mediate the conversion of CD4^+^ T cells [174,175]. Tregs promote immune suppression by secreting TGF-β and IL-10 [176]. Contrarily, Tregs are associated with a good prognosis in CRC [177]. Th17 is another type of helper T cell induced by TGFβ- and IL-10. Th17 secretes IL-17, a pro-inflammatory cytokine which mediates tumorigenic and angiogenic effects. The frequency of IL-17 positively correlates with tumor recurrence in HCC, CRC, and pancreatic intraepithelial neoplastic cells [178,179,180]. Neutrophil recruitment induced by IL-17 aggravates nonalcoholic steatohepatitis via fatty acid release, resulting in HCC [181]. By contrast, Amicarella et al. [182] reported that IL-17 promotes the recruitment of cytotoxic CD8^+^ T cells and beneficial neutrophils via CCL5-CCL20 and IL-8, respectively. The cytokine-induced killer (CIK) cells are subset of CD8^+^ T cells derived from human peripheral blood lymphocytes. CIKs are expanded in vitro using IL-2, IFN-γ, and anti-CD3 antibodies. Other reported sources of CIK cells include the bone marrow and umbilical cord [183,184]. CIK cells are classified into two subsets, CD3^−^CD56^+^ and CD3^+^CD56^−^, due to their expression of CD16 and CD56 [185] and CD3^+^CD56^+^, which perform the effector function. CIK cells attack cancer cells via the Fas ligand [186] and NK-activating receptors such as NKG2D, NKp30, and NKp46 [187,188]. CIK cells serve as a form of adoptive T cell therapy (ACT), whereby a patient’s own peripheral blood mononuclear cell is used to expand anti-tumor CIK cells that are subsequently reinjected back into patients. Donor CIK cells are also used as alternatives in cases where a patient’s own CIK cells are insufficient.

#### 3.5.2. B Lymphocytes

B lymphocytes are critical for humoral immunity by killing invaders at a distance. B lymphocyte consists of three subsets: B1 lineage, MZ (marginal zone) lineage, and FO (follicular) lineage. B lymphocytes have the ability to present antigenic peptides to CD4^+^ and CD8^+^ T cells [189]. Several studies have shown that B lymphocytes express cytokines and co-stimulatory molecules primarily for T cell activation, such as IFN-γ, BCL-6, CD 40L, and CD28 [190,191,192]. However, they are not as potent as DCs. In addition, they express TLRs, which are activated by pathogen-associated molecular patterns [192]. Controversy exists regarding the exact role of B lymphocytes in solid tumors. They have been reported to promote tumor growth by inhibiting CD4^+^ T cell priming and the cytotoxic effect of CD8^+^ T cells in B cell-deficient mice [193]. Similarly, hepatic B lymphocytes have been found to downregulate surface co-stimulatory CD80 expression through interactions with intrahepatic MDSCs in a colorectal liver metastasis model, leading to impaired T cell activation [194]. This can be reversed in the absence of MDSC through STAT3 signaling, which is the key factor regulating MDSCs. B cells have also been implicated in promoting an immunosuppressive milieu through secretion of IL-10 in HCC [195]. Another study showed that B lymphocytes can switch and convert to B-reg phenotypes [196]. A higher B lymphocyte-activating factor correlates with disease progression in HBV-HCC, indicating poorer median survival compared with non-HBV-HCC and non-HCC controls [197]. In PDAC, a subset of B lymphocyte CD5^+^ secretes IL-35 which promotes tumor growth [198]. Gunderson et al. [199] found that B cells promoted macrophage polarization to an immunosuppressive phenotype and further confirmed the pro-tumorigenic role of B cells by transplanting PDAC cells into B-cell-deficient mice. Conversely, the amount of B lymphocytes present in HCC has been reported to correlate with T cell activation markers, indicative of a close interaction between both T- and B-cells and a better prognosis [200]. Furthermore, B cell-depleted mice indicates that B cells might be critically involved in decreasing tumor growth in established HCC, while the prevention of initial tumor formation is a role played by T cells [201]. Similarly, the infiltration of CD20^+^ B cells in metastatic CRC has been reported to improve survival rates [202].

#### 3.5.3. NKT Cells

Natural killer T cells (NKTs) are subset of T cells that possesses an antigen-specific T cell receptor (TCR) that recognizes self and foreign antigens, specifically lipid antigens i.e., α-galactosyl ceramide (α-GalCer) that are undetected by CD8^+^ and CD4^+^ T lymphocytes. NKTs can be iNKT (Type I NKT cells), with an invariant TCR, or Type II NKTs (variable or diverse TCR). Invariant NKTs have been reported to produce anti-tumor factors such as IFN-γ and activate both CTL and NK cells [203], whereas variable NKTs inhibit anti-tumor immunity. Contrasting results regarding the role of iNKT cells have been reported in gastrointestinal tumors, especially CRC. For instance, an abundance of NKT cells in HCC has been reported [204,205]. The profibrotic role has also been reported in the non-alcoholic steatohepatitis (NASH) model of HCC [205]. By contrast, a study showed that a strain of gut microbiome (*Clostridium* species) uses bile acids as a messenger to regulate CXCL16 levels in LSECs, thereby increasing CXCR6^+^ hepatic NKTs. The accumulated NKTs inhibit tumor growth in primary and metastatic liver tumors [206]. Similarly, the absence of NKTs is associated with increased pancreatic tumor development and progression in LSL-Kras^G12D/+^ mice. The pharmacological inhibition of arachidonate 5-lipoxygenase (5-LOX) and microsomal prostaglandin E synthase-1 (mPGES-1) led to reversal of the NKT population, enhanced CD8^+^ T cells, and tumor suppression [207]. Moreover, higher Valpha24^+^ NKT cell infiltration in colorectal carcinomas has been reported as an independent factor for favorable prognosis [208]. By contrast, evidence regarding the role of NKTs in intestinal inflammation in both human and animal models of ulcerative colitis suggests that NKTs may favor tumor development in intestinal tissue. Wang et al. [209] investigated the iNKT-mediated regulation of tumor immunity in an orthotopic spontaneous model of the early stages of intestinal cancer in Apc ^min/+^ mouse model of CRC deficient of all NKT and/iNKT cell. The study found a reduction in intestinal polyps, an increased proportion of FOXP3 Tregs, and a reduced expression of genes associated with TH1 in the absence of iNKT. In the presence of iNKTs, an enhanced proportion of M2 macrophages and MDSCs was found in the presence of iNKT. Consistently, Heller et al. [210] that reported the pro-inflammatory role of iNKTs in a mouse model of intestinal disease.

#### 3.5.4. Regulatory T Cells

Tregs are characterized by their ability to activate the FOXP3 gene. Tregs release cytokines such as IL-10, CTLA-4, and TGF-β, and exerts an inhibitory effect on both B- and T-lymphocytes (see Figure 4). It has been shown that Tregs may have an influence on macrophages (M1/M2) and neutrophils (N1/N2) phenotype [211]. Tregs also promote the destruction of their target cells by releasing granzymes and perforins. The high prevalence of Treg CD4^+^CD25^+^FOXP3 correlates with the poor prognosis and recurrence of solid tumors, including HCC and PC [212,213]. However, the role of Tregs in CRC remains controversial. As opposed to the immunosuppressive role of Tregs, numerous studies have shown that the high intra-tumoral frequency of Tregs correlates with good prognosis in patients with CRC [214,215,216]. By contrast, Tregs within the same type of tumor suppress T cell response by promoting recurrence [217]. The alternative role of Tregs in CRC may be due to changes in the immune microenvironment that causes the expression of RORγt by Tregs, which led to a switch from an anti-inflammatory to inflammatory role through IL-17 secretion [218]. Additionally, another study showed that Foxp3+ Tregs are enriched with of RORγt in mouse colon and lymphoid tissues [219,220].

## 4. Immune Landscape Determinants and Predictors in GI Cancer

**Figure 5:**
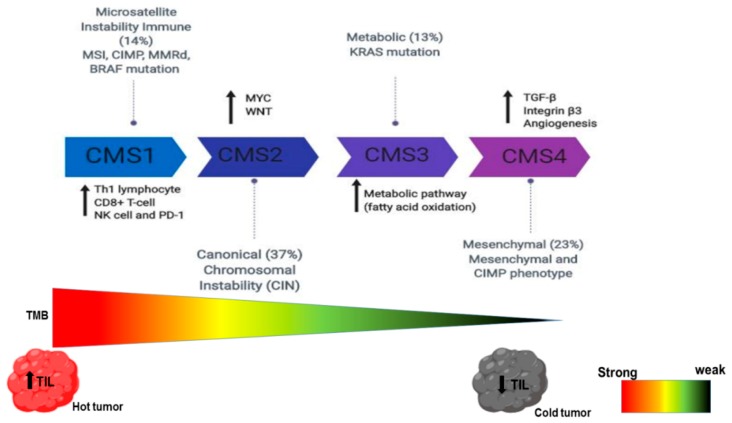
The consensus molecular classification (CMS) of CRC in correlation with tumor infiltrating lymphocyte (TIL) abundance. CMS1 is infiltrated with T helper 1 lymphocytes, CD8^+^ T cells, NK cells, PD-1, tumor mutation burden (TMB) and increased TIL as compared with other CRC subtypes. High TIL infiltration represent a hot tumor, which can be targeted with an immune checkpoint inhibitor, whereas reduction in TIL denotes a cold tumor.

Molecular subtypes and stratification identify the drivers of human cancer. This strategy has been used to profile the molecular, genetics, and immune composition of cancer, while an appropriate treatment regimen is assigned [221,222]. Notably, there have been a recent breakthrough using high-throughput next generation sequencing and consensus-based clustering technique to map the immune landscape to the genetics of tumors. Remarkably, CRC has been successfully stratified, and a consensus on four molecular subtypes (CMS) has been reached (see Figure 5).

**Figure 6:**
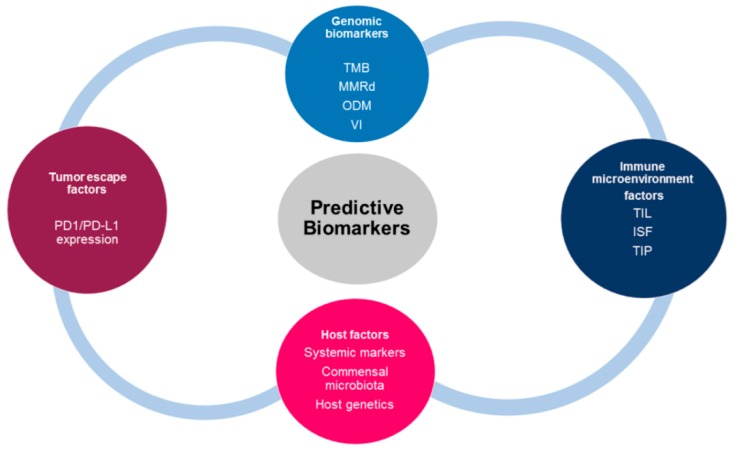
Emerging predictive biomarker of response to immune checkpoint inhibitor. The programmed cell-death 1 and its ligand (PD-1/PD-L1) are tumor escape factors used widely as predictive biomarkers for patient’s response to immunotherapy. Other suggestive biomarkers include genomic, tumor-escape, immune-microenvironment and host factors. These factors include TMB (tumor mutation burden), MMrd (mismatch repair deficiency), VI (oncogenic viral infection), ODM (oncogenic driver mutation), TIL (tumor infiltrating lymphocyte), ISF (Immune suppressor factor) and tumor immune phenotype (TIP).

CMS1 denotes microsatellite instability subtypes that are composed of microsatellite instability (MSI), CpG island methylator phenotype (CIMP), mismatch repair deficiency (MMrd), and BRAF mutations; CMS2 denotes canonical or epithelial subtypes consisting of chromosomal instability; CMS3 denotes a metabolic subtype consisting of KRAS mutations; and CMS4 denotes a mesenchymal subtype consisting of mesenchymal and CIMP phenotypes [223]. By contrast, there is little consensus on molecular subtypes in both pancreatic ductal adenocarcinoma (PDAC) and HCC, as these are still in their infancy. Similarly, tumors have also been classified based on tumor-infiltrated lymphocytes (TILs) and macrophages. The immune-inflamed or hot tumors are characterized by the high infiltration of immune cells; the immune-excluded is identified and characterized by immune cells which are trapped at the boundaries of the tumor; and immune-desert or cold tumor phenotypes are characterized by a complete absence of immune cells [224,225]. However, the limitation of the classification is that most studies have not simultaneously considered the boundaries and tumor core. The success of programmed cell death-1 and its ligand (PD-1/PD-L1) has been demonstrated in many cancers [226,227]. Nevertheless, some patients do not derive benefits from the immune checkpoint inhibitor (ICI). For instance, the failure of nivolumab in a phase III study has been reported [228,229], despite its success alongside pembrolizumab in phase II clinical trials [230,231]. Furthermore, a trial testing pembrolizumab in combination with dexamethasone (immunomodulatory agent) with either lenalidomide or pomalidomide in the treatment of patients with multiple myeloma was terminated due to death risk (US Food and drug administration alerts, 2018). An effective predictive biomarker is therefore needed to stratify patients who may respond to an immune checkpoint inhibitor. Several mechanistic studies have identified the determinants of tumor immune contextures as biomarkers for predicting the patient’s response to immune checkpoint inhibitors (see Figure 6).

**Figure 7:**
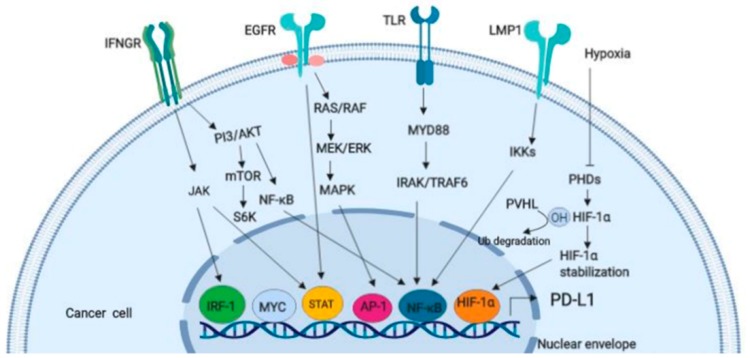
Signaling pathways for PD-L1 expression in cancer. Programmed death ligand-1 (PD-L1) is a cell surface receptor on cancer cells modulated by interferon gamma receptor (IFNGR), epidermal growth factor (EGFR), toll-like receptor (TLR), Epstein–Barr virus latent membrane protein (LMP1) and hypoxia.

PD-L1 is critical in predicting patient’s response to PD-1/PD-L1 inhibitor immunotherapy. Thus, PD-L1 expression using immunohistochemistry is the only approved biomarker widely employed to distinguish responders from non-responders [232]. However, there are limitations with the use of PD-L1 as a predictive biomarker. First, there have been contradictory results on the association between PD-L1 expression and immune checkpoint efficacy [233]. Furthermore, PD-L1-negative patients benefit from immune checkpoint inhibitor therapy [234]. Other limitations include non-standardized cutoff to access PD-L1 positivity, unreliable predictive ability in CRC and HCC [235], different detection techniques [236], and the types of cells expressing PD-1/PD-L1 molecule within the tumor [237]. Thus, it can be concluded that PD-L1 expression is not a one-size-fits-all biomarker. TIL is another predictor of immune checkpoint inhibitor efficacy and also a strong prognostic biomarker for some cancers regardless of the ICI. For example, the tumor-inflamed phenotype exhibits a better response to ICI due to the high infiltration of TILs within the stroma [238]. Surprisingly, some patients with a tumor-inflamed phenotype still do not derive benefit. A study showed that the early infiltration of regulatory T cells (Tregs) may hinder the cytotoxic effect of TIL [239]. It has been reported that mutations within cancer cells encode for neoantigens, recognized by T cells when presented by the MHC molecules [166]. In addition, the number of non-synonymous single nucleotide variants (nsSNVs), somatic copy numbers, and indels are hypothesized to be contributors to the tumor mutation burden (TMB). A high TMB observed in CRC and HCC positively correlates with PD-1/PD-L1 expression, whereas PDAC has the lowest TMB [240,241]. Conversely, a meta-analysis by Liu et al. [242] found no association between PD-1/PD-L1 and TMB in curative resected HCC. Evidence from both tumor samples and patients implicates V-domain Ig suppressor of T cell activation (VISTA) as the main inhibitory checkpoint expressed in PDAC [243]. Indeed, the elevated mutational burden and expression of cytotoxic T cells, NK cell infiltration, Th1 lymphocytes, and PD-1 due to enhanced neoantigens are hallmarks of the CMS1 subtype of CRC [221] (see Figure 5). A mutation per mega base of 17 or above has been reported to correlate with microsatellite instability (MSI-CRC) in CMS1 of CRC [244], although other studies have reported a variable TMB cutoff point [245]. Remarkably, 3% of microsatellite stable (MSS-CRC) patients also benefit from a high TMB cutoff [246]. Similar results identify a correlation between TMB and immune checkpoint response in many cancer types [247]. Surprisingly, some patients with a high TMB do not respond to the immune checkpoint inhibitor and vice versa [248,249]. Contrary to reports on TMB and ICI response, a recent study utilized the quanTIseq deconvolution approach on 8000 cancer genome atlas (TCGA) tumor samples across 19 solid tumors and found that both mutation burden and tumor clonality heterogeneity are weakly associated with infiltrated CD8^+^ T cells [250]. However, this study reported a stronger association between CXCL9/CXCR3 and CD8^+^ T cell infiltration. Tumor clonality also influences the response to the immune checkpoint inhibitor but does not contribute to mutational burden. Clonal mutation from homogenous tumors has been reported to enhance immune response more effectively than subclonal mutation from heterogeneous tumors [251]. Notably, MSI is a type of mismatch repair deficiency (MMRd) which generates indel and frameshift mutations. This stimulates neoantigens, leading to increased T cell infiltration with an improved response to the immune checkpoint inhibitor [252]. However, MMRd gives rise to subclonal neoepitopes that are less effective at generating a good response to ICI [251,253]. The mechanism by which MMRd generates a better response to ICI is not understood. The loss of tumor suppressor genes (TSG), oncogenic driver mutations, and oncogenic pathways (see Figure 7) have also been found to contribute to immune composition and immune checkpoint response.

Research has shown that driver oncogenes or a loss of TSG enhance immune suppression and attenuate immune responses in GI cancers. For example, a mutation in TP53 is associated with increased immunosuppressive cells (Tregs and T follicular helper) and immune checkpoint molecules (CTLA-4, PD-1 and T-cell immunoglobulin and mucin domain-containing protein 3 (TIM-3) in HCC [254]. P53 loss decreased cytotoxic CD8^+^ T cells and increased Th1/2 shifts, which promote gemcitabine resistance, thus leading to a reduced survival in PC mouse models [255]. Similarly, mutation in P53-induced TAM population and Tregs leads to tumor progression [256]. Another study showed a positive correlation between P53 loss or KRAS mutation alone or co-occurrence of both mutations and ICI response was reported in lung adenocarcinoma [257]. Consistently, PD-L1 expression is higher and correlates with P53 mutation in NSCLC than in CRC [258]. In addition, a positive correlation between KRAS mutation and PD-1/PD-L1 was found in PC [259]. Contrarily, a lower efficacy in response to anti-PD-1 was reported in CRC with a KRAS mutation [260]. Other clinical studies on NSCLC reported that epidermal growth factor receptor (EGFR) mutations and anaplastic lymphoma kinase (ALK) rearrangements, KRAS, and BRAF do not influence the efficacy of the immune checkpoint inhibitor [261,262,263]. Furthermore, Balli et al. [248] reported an inverse correlation between NOTCH2-, MYC-, and fibroblast growth factor receptor-1 (FGFR1)-amplified tumors and cytolytic T cell response rather than mutation load in pancreatic tumors. Further research has revealed the modulatory effect of microbiota on the efficacy of ICI. For instance, Sivan et al. [264] identified a relationship between *Bifidobacterium* and the PD-1 inhibitor in a mouse model of melanoma. *Bifidobacterium* enhanced anti-PD-1 efficacy by augmenting dendritic cell function and CD8^+^ T cell priming. Similarly, patients with *Faecalibacterium* had longer progression-free survival and higher TILs compared with patients who had a lower abundance of this strain of bacteria [265]. Furthermore, the over-presentation of *Ruminococcaceae, Faecalibacterium*, and *Clostridiales* in peripheral blood positively correlates with CD8^+^ T cells while Bacteroidales correlates with MDSCs and Tregs [265]. Routy et al. [266] compared clinical outcomes when patients were treated with the PD-1 inhibitor in combination with or without antibiotics. Treatment with antibiotics and PD-1 resulted in shorter overall survival and progression-free survival alone or with the CTLA-4 inhibitor. By contrast, bacterial ablation upregulated T cell and PD-1 expression in a mouse model of PDAC [16]. As opposed to PD-1, CTLA-4 efficacy is determined by *Bacteroides fragilis*. The colonization of *B. fragilis* in germ-free mice improved the anti-tumoral response of anti-CTLA-4 therapy [267]. Other emerging predictive biomarkers include immune microenvironment factors, host factors (see Figure 6).

Additionally, the carcinoembryonic antigen (CEA) and carbohydrate antigen 19-9 (CA19-9) are glycoproteins and cancer antigens mostly found elevated in solid tumors [268]. CEA and CA-19-9 are widely used as the prognostic and/or predictive biomarkers for the detection and management of adenocarcinoma including GI cancer. A recent study showed that serum CA-19-9 predicts PC in acute pancreatitis patients [269]. Similarly, CEA level is a recommended prognostic biomarker according to the national comprehensive cancer network (NCCN) guidelines in CRC. Based on the sensitivity, the clinical use of these prognostic biomarker is controversial. Notably, these cancer antigens are prerequisite for clinical therapy design for bispecific T cell engagers (BiTES) (CD3*CEA), anti-CEA Chimeric antigen receptor T cell (CAR-T), anti-epithelia cell adhesion molecule (EPCAM) CAR-T and several others. These are currently being tested for their efficacy in clinical trials, whereas some of them showed promising result in clinical trials (as discussed in Section 5).

## 5. Progress and Current Immunotherapeutic Approaches in GI Cancers

### 5.1. Adoptive T-Cell Therapy 

#### 5.1.1. Chimeric Antigen Receptor T Cells

The chimeric antigen receptor T cell (CAR-T) is a major breakthrough in oncology. CAR-Ts are genetically engineered and designed to recognize tumor antigens in the absence of MHC presentation through the expression of CARs. The CARs fuses an antibody-binding domain to the signaling domain linked to the co-stimulatory domain [270,271]. The only approved CAR-T is anti-CD19 for the treatment of B-cell lymphoma, which targets the co-stimulatory molecule CD19 in B-cell lymphoma and leukemia [272]. However, a tolerable side effect may occur as well as relapse due to CAR-binding epitopes [270,273]. Other limitations from CAR-T treatment include exhausted CAR-T and poor trafficking into the tumor [274,275]. Several attempts to treat solid tumors with CAR-T therapy have been unsuccessful due to the nature of the tumor microenvironment as well as contributing factors such as hypoxia, which leads to toxicity in treated patients [276,277,278]. Furthermore, evidence from pre-clinical studies has identified loopholes, and strategies that aim to address setbacks from CAR-T treatment in solid tumors have been extensively discussed [279,280,281]. Notably, therapeutic approaches such as combination strategies and the modification of CAR-T have been implemented. Several of them are undergoing clinical trials (NCT02729493, NCT03323944, NCT03638206) (see Table 1).

For instance, a recently completed phase 1 clinical trial of anti-glypican-3 (GPC3) CAR-T, evaluating its efficacy and safety for 13 patients with R/R (relapse/refractory) HCC, yielded promising results. All patients tolerated the treatment; one patient had a serious adverse event (SAE) grade 3 fever. Patients treated without lymphodepletion conditioning had progressive disease after receiving anti-GPC3 CAR-T. Conversely, treatment with lymphodepletion conditioning results in one partial response—three patients had a stable disease (SD), two had a progressive disease, and one death was reported in the stable disease group after 108 days [282]. Another completed phase 1 trial showed that CEA CAR-T was well tolerated in CRC patients. Seven patients who previously had progressive disease had a SD, with two of the seven patients having a SD for 30 weeks. Two patients had tumor shrinkage, and CEA was reduced in the blood of most patients. Lastly, CAR-T was found to proliferate and persist in the patient’s blood after the second infusion [283]. (see Table 2).

A different CAR-T approach has recently been developed that can eliminate cancer cells with ET1402L1 T cells in HLA-A* 02:01 restricted AFP^+^ in HCC [293]. This approach has been found to counteract tumor growth in pre-clinical studies and is currently being tested for safety and efficacy in ongoing trials (NCT03888859). However, CAR-T therapy such as anti-mesothelin CAR-T (NCT01583686) in metastatic PC has been terminated for unknown reasons.

#### 5.1.2. Tumor-Infiltrating Lymphocytes

TILs are employed in ACT, a procedure in which T cells are derived from resected metastatic tumor or blood and expanded in vitro in the presence of IL-2, anti-CD3 antibodies, and irradiated autologous or allogenic feeder cells [294]. The final product is infused into lymphodepleted patients to inhibit tumor progression [295,296]. The modification of the tumor-reactivity selection step results in the so called “young TIL protocol,” which is a shorter process [297]. Recent advances involve high-throughput genetic sequencing to identify the non-synonymous mutation and subsequent synthesis of mutation peptide pulsed with DCs. The isolated TILs from the tumor are co-cultured with pulsed DC peptide, recognized by the subsequent expansion of T cells [298]. A higher percentage of TILs within tumor predicts survival in many cancer patients, including those with GI cancer [299,300]. Tran et al. [301] found that TILs derived from nine out of 10 patients in metastatic GI cancer contained CD4^+^ T cells and CD8^+^ T cells that recognize one to three neoepitopes from somatic mutation. In addition, TILs adoptive cell therapy yielded 50% ORR and 13% complete response (CR) in melanoma patients [296], which surpass responses from immune checkpoint inhibitors in reported cases. Even the most difficult forms of breast cancer; triple negative breast cancer (TNBC) and human epidermal growth factor (HER2) was successfully treated with TILs [302,303]. Promising results have also been reported in cholangiocarcinoma after infusion with CD4^+^ T cells that recognized the Erbb2 binding protein ERBB2IP epitope [304] and HLA-C*8:02 in restricted KRAS G12D lung metastasis CRC [305]. Interestingly, CD8^+^ neoantigen T cells specific to SMAD5 and mucin-4 (MUC4) epitopes, CD4^+^ and CD8^+^ memory T cells specific to KRASG12D and KRASG12V, respectively, have been isolated from peripheral blood of epithelial cancer patients [306]. Future clinical trials will test neoantigen-specific T cells and TCRs for the P53 mutation in metastatic cancer [307]. Despite the efficacy of TIL therapy, the limitations of this approach include the poor survival of T cells, the poor efficiency of the method used to detect neoantigen reactive T cells, and the derivation of terminally differentiated exhausted T cells that express immune checkpoint protein [308,309]. More recent studies have reported strategies that can improve the efficacy of TIL treatment, such as reprogramming exhausted differentiated T cells using the Sendai virus to transduce octamer binding protein-3/4 (OCT3/4), sex determining region-Y2 (SOX2), kruppel-like factor (KLF4), and c-MYC to induced pluripotent stem cell (iPSC) [310]. In addition, the enhancement of neoantigen reactive T cells through the enrichment of PD-1-expressing T cells by using a micro well culture method to prevent the overgrowth of non-reactive T cells [298]. The prevalence and application of TILs in targeting KRASG12D in patients with gastrointestinal cancer have been a subject of debate [311]. Treatment with TILs is currently undergoing clinical trials (see Table 1), and the results are eagerly awaited.

#### 5.1.3. CIK

The efficacy of cytokine induced killer cells (CIK) therapy has been demonstrated in gastrointestinal tumors such as CRC, HCC, and PC. In addition, combination therapy has also been reported to enhance efficient cytotoxic and anti-tumor activity of CIK. Consequently, CIK has been used in combination treatment with dendritic cells (DCs), cytokines, immune checkpoint inhibitors, chemotherapy, chimeric antigen receptors, antibodies, and nanoparticles. These combination strategies have produced encouraging results. For instance, pre-clinical and clinical studies have shown that CIK in combination with DCs exhibited better efficacy than CIK monotherapy in HCC and post-trans catheter arterial chemoembolization (TACE) HCC [312,313,314]. A study on 67 patients reported partial remission in five patients and a stable disease in 29 patients with a decreased migration and proliferation in cancer cells. Cytokine induced killer cells-dendritic cells(CIK-DC) combined with sorafenib also improved patients’ overall survival rate with no adverse effect in advanced HCC [315]. Similarly, CIK-DC also showed improved overall survival of five years and disease-free survival in CRC [316,317,318]. The transfusion of autologous CIK and a meta-analysis of DC-CIK showed an improved overall survival rate for advanced PC [319,320]. Furthermore, the clinical efficacy of adjuvant therapy with activated CIK treatment showed an increased median recurrence-free survival (RFS) time of 44 months compared with the reccurrence free survival (RFS) of patients receiving curative treatment for liver cancer [321]. Several clinical trials testing the efficacy and safety of CIK-DC in combination with immune checkpoint inhibitors, chemotherapy, and others are currently ongoing. One of these will examine the outcome of combination therapy with CIK-DC plus Anti-PD-1 in refractory solid tumors, including HCC and CRC (NCT02886897); the efficacy and safety of adjuvant CIK in HCC patients undergoing liver transplantation (NCT03983967); and intermediate stage HCC who have undergone TACE (NCT02856815).

### 5.2. Bispecific T-Cell Engagers

Bispecific T cell engagers (BiTEs) that recognize cancer stem cell (CSC) antigens (CD19) and antigen-binding domains of antibodies (CD3) attract CSCs while promoting T cell-induced cytotoxicity as well as redirecting the effector function of a number of immune cells [322,323]. The only approved BiTE is Blinatumomab, which is given as a continuous infusion and results in a rare remission rate in patients [323]. Phase 1 clinical trials of EpCAM/CD3 T cell engager (Solitomab) in solid tumors have shown an anti-tumor effect with a significant side effect [290]. Furthermore, a phase 1 study of CEA*CD3BiTES was abruptly terminated due to the detection of BiTES antibodies in patients treated with a high dose, with disease progression in 73% of patients with advanced gastrointestinal cancer [324].

### 5.3. Cancer Vaccines

Cancer vaccines are developed to specifically target tumor-associated antigens (TAAs), virus-associated antigens, cancer germline antigens, or tumor-specific antigens (TSAs) (neoantigen). Despite treatment with an immune checkpoint inhibitor, cancer-specific immune responses remain suboptimal, leaving options for alternatives and new therapeutic approaches. Neoantigens expressed by mutated cancer cells can generate optimal CD4^+^ and CD8^+^ T cell responses [325]. A recent study reported the stimulatory effect of a peptide neoantigens vaccine on CD4^+^ rather than on CD8^+^ T cell responses [326,327]. Remarkably, a neoantigen-based vaccine that contains up to 20 neoantigen peptides that simultaneously targets multiple proteins showed promising results [328,329]. However, a study showed that the multipeptide vaccine failed to improve survival rates [330]. Similarly, a DNA vaccine that targets enolase-1 (ENO1) in genetically engineered mice with PDAC showed promising results but did not completely eradicate tumor growth [331]. Nevertheless, combination strategies have been shown to improve the efficacy of the ENO1 DNA vaccine in animal model. In addition, a multipeptide HCC vaccine IMA970A (HepaVac-101) with a CV8102 adjuvant is currently being tested in phase I/II clinical trials for very early and intermediate stage HCC positive with HLA haplotype (NCT03203005). The vaccine is composed of 16 peptide cocktails, with seven peptides restricted to HLA-A*02, five to HLA-A*24, and four to HLA class II. Of particular clinical relevance was a recent finding that the replication-deficient human type 5 recombinant adenovirus (Ad5) vaccine encoding guanylyl cyclase C (GUCY2C) fused to the PAn DR Epitope (PADRE) (Ad5-GUCY2C-PADRE) vaccine yielded positive results in the phase 1 clinical trial of early CRC patients [332]. The vaccine stimulated optimal CD8^+^ T cell responses, while CD4^+^ T cells were eliminated by self-tolerance, a condition known as “split tolerance” in the absence of neutralizing antibodies to the viral vector. Oncolytic viruses (OVs) are genetically engineered DNA viruses. OVs selectively infect highly replicative tumor cells by lytic cell destruction and dendritic cell activation through GM-CSF to stimulate T cell responses. OVs include adenoviruses, herpes simplex viruses, vaccinia virus, and vesicular stomatitis virus. OVs activate retinoic acid inducible gene -1/stimulator of interferon gene (RIG-1/STING) and TLR pathways [333,334]. Therefore, STING and TLR agonists are now being tested in clinical trials alone or in combination with immunotherapies. The oncolytic virus T-vec has been shown in all phases of clinical studies to be tolerable for patients with various types of cancer including GI cancer, and hence, its approval in melanoma [335,336,337,338]. Additionally, OV in combination with the CTLA4 blockade has been shown to be effective in melanoma [339]. By contrast, a combination approach with chemotherapy (gemcitabine) showed limited efficacy in oncolytic adenovirus-treated PC (NCT02045589) [340]. Furthermore, a recent study showed that OV Reo inhibits viral-induced oncogenic drive and tumor mutation burden in hepatitis C virus (HCV)-HCC [341]. A phase 1 study assessing the safety of an autologous cancer vaccine showed no significant side effects in 30 advanced solid tumors, including CRC. The study reported 23 SD out of 27 and one progressive disease (PD) after first vaccination [342]. Finally, small anticancer molecules have also been used as a potent sensitizers of tumor cells for the improvement of oncolytic potential [343].

### 5.4. Checkpoint Inhibitors

Immune checkpoint inhibitors have been used successfully in treating a wide range of cancers with the exception of GI cancers [344,345]. Checkpoint molecules serve as “brakes” which inhibit the cytotoxic effect of T cells. The first approved checkpoint inhibitors are ipilimumab, an inhibitor of CTLA-4, and prembolizumab, which inhibits programmed cell death protein 1 (PD-1) [346,347]. Recently, several studies have focused on critical components and immune contexture by tumor stratification in predicting therapeutic outcomes across various cancers. For instance, tumors with a high mutation burden are better targets of immune checkpoints due to the highly expressed tumor antigen [223,348]. Despite the enhanced mutational burden, some patients remained unresponsive to immune checkpoint therapies [248]. Several mechanistic studies have identified the involvement of multiple immunosuppressive pathways [349,350]. Hence, the therapeutic blockade of TIGIT, VISTA, LAG-3, and TIM-3 are therefore now under investigation. Several strategies are currently piggy-backed on the success of checkpoint inhibitors [325]. Hence, the use of a combination therapy with checkpoint inhibitors (see Table 3).

Currently, ongoing studies are testing the safety and efficacy of immune checkpoint inhibitors and sorafenib in advanced HCC, whereas gemcitabine in combination with checkpoint blockade PDL-1 is being evaluated for the treatment of PC. Furthermore, combinations of radio frequency ablation, cyoablation, or TACE and CTLA-4 inhibitor in advanced HCC shows promising results [351], and phase 2 is currently ongoing. Notably, a combination of regorafenib and nivolumab (anti-PD-1) in patients with advanced CRC yielded a 29% response rate, a reduced FoxP3hiCD45RA-Tregs fraction, a manageable safety profile, and an anti-tumor effect [286]. By contrast, phase 3 studies on the combination of cobimetinib or atezolizumab plus regorafenib failed to improve overall survival rates, and three treatment-related deaths was reported [287].

### 5.5. NK Cell-Based Therapies

NK cell express inhibitory molecules such as KIR (killer cell immunoglobulin-like receptor), NKG2A, and TIGIT. Similarly, a subset of NK cells has been reported to express checkpoint molecule (PD-1) [352]. Monoclonal antibodies targeting these molecules are currently being tested alone or in combination with other immunotherapeutic therapies [353,354]. Potent stimulators of the cytotoxic effect of NK cells includes IL-2 and IL-15. Allogeneic NK cell transfer is widely used in clinical trials due to its promising results [355]. For instance, a study reported that irreversible electroporation in combination with allogeneic NK cells improves the median overall survival in stage IV HCC patients [356]. Similarly, the combination of irreversible electroporation and allogeneic NK cell increases progression free survival and overall survival in stage III/IV PC [357]. Another study showed that combination of either 5-fluorouracil or oxaliplatin with adoptive NK cell increases five years’ progression free survival and overall survival with no severe adverse effect in CRC [358]. A phase 1 study testing the combination of trastuzumab or cetuximab with adoptive NK cell therapy is well tolerated with four stable diseases; three of which show a decreased tumor size, and two out of the six treated patients had progressive diseases [359]. Furthermore, the use of CAR-NK in solid tumors is still in its infancy, as most studies are from preclinical studies and early clinical trials. Pre-clinical studies of CAR NK-92 targeting receptor tyrosine-protein kinase erbB-2 (ErbB2)/HER2 and EGFR showed promising results in glioblastoma and renal carcinoma lung metastases, respectively [360,361]. CAR-NK are currently being tested for their safety and efficacy, and the use of modified cytokines in combination with allogeneic NK is currently ongoing (NCT02890758).

### 5.6. Stroma-Targeted Therapies

The stroma is a critical component of a tumor that encompasses the extracellular matrix, recruited mesenchymal stromal cells, fibroblasts, osteoblasts, chondrocytes, macrophages, and myeloid cells, which contributes to cancer progression [362,363]. Anti-FAP antibodies conjugated to drugs induces a cytotoxic effect, which inhibits tumor progression in gastrointestinal cancer [364]. Less specific strategies involve the use of FAP to cleave and activate pro-drug promethelin, as well as the methelin released exerts cytotoxic effect [365]. In addition, the use of both single-agent CAR-T as well as combined agent anti-tumor CAR-T specific for antigen erythropoietin-producing HCC A2 (EphA2) on FAP^+^ CAF were reported [366,367]. A study showed that the anti-FAP CAR-T and DNA vaccine inhibits tumor growth in a mouse model of GI cancer [368,369]. In addition, a FAP-specific vaccine induces cytotoxic T cells and also promotes chemotherapy sensitivity [368,370]. CAF-derived TGF-β plays an inhibitory role on CD4^+^ and CD8^+^ T cells [367,371]. Therefore, the simultaneous blockade of TGF-β and PD-L1 facilitates T cell infiltration into the tumor microenvironment [91]. However, TGF-β inhibitors failed to improve the efficacy of anti-PD-1 in tumors [372]. The proliferation of CAF triggered by TGF-β inhibition increases matrix metallopeptidase 9 (MMP-9) and reduced the expression of PD-L1. Therefore, a synergetic and sequential approach has been suggested that involves delaying TGF-β inhibition until anti-PD 1 resistance is observed [373,374].

Cytokines and chemokines act in a paracrine and autocrine manner within the tumor microenvironment. For instance, IL-8 promotes immunosuppression and tumor escape. In addition, the serum level of IL-8 is associated with a poor prognosis in patients [375]. Furthermore, cytokines are known to have shorter half-lives, and their systemic use is limited due to toxicity [376,377]. Of a clinical benefit was the recent advances made towards modification of cytokines by the attachment of polyethylene glycol (PEG) to prolong their shelf lives. Modified cytokines include pegilodecakin (IL-10) (AM0010), biomimetic IL-2 pulmoleukin, IL-2 variant (IL-2v) linked to anti-FAP, and ALT-803; and others are still in development. For example, NKTR-214 (bempegaldesleukin) can target interleukin-2 (IL-2) receptor beta subunit with the potential of turning cold tumors to hot tumors. Most cytokine-based drugs are now given in combination with checkpoint inhibitors, chemotherapy, and CAR-T to enhance their efficacy [378]. Clinical trials testing the efficacy are currently ongoing (NCT03400332) (see Table 3). Similarly, a study showed that inhibition with anti-CXCR2 and anti-PD-1 synergistically inhibits tumor formation [379]. Other stroma-targeted strategies include CSF-1R, CXCR1, CXCR2, and CCR2/CCR5 blockades, which inhibit the trafficking and recruitment of myeloid cells such as macrophages into the tumor [380,381]. Surprisingly, CSF-1R inhibitors have failed to improve survival rates in early clinical trials. A recent phase 1 study of emactuzumab alone or in combination with paclitaxel in advanced tumors showed a 7% ORR without a relevant anti-tumor effect [292]. By contrast, pre-clinical studies showed that CSF-1R inhibition enhances PD-1 in a melanoma mouse model with a BRAF mutation [382].

## 6. Conclusions

GI cancer is a pervasive disease with pathogenic contributions. The tumor immune-microenvironment play a role in the pathogenesis. Mounting evidence that has accumulated from preclinical studies over the years has identified immune therapeutic targets. However, the overall survival advantage of patients treated with immunotherapies in solid tumors including GI cancer is suboptimal. Recent advances in predictive/prognostic biomarkers and combination treatment approaches have been made towards improving patients’ responses to immunotherapy. The understanding of the tumor-immune microenvironment and identification of potential predictive biomarkers could hold the answers to future immunotherapy targeting the GI cancers, in particular for the development of precision-based medicine.

## Figures and Tables

**Table 1:** Clinical trials of various adoptive immunotherapies in gastrointestinal (GI) cancer.

Cancer Type	Start Year	No of Participants	Site of Trial	Agents	Phase	Clinical Trials Gov Number	Recruitment Status
**CAR-T Therapies**							
Liver neoplasms	2015	25	China	EPCAM CAR-T cells	N/A	NCT02729493	Yes
Metastatic cancer	2012	15	US	Mesothelin CAR-T	I/II	NCT01583686	Terminated
HCC	2018	50	China	c-Met/PD-L1 CAR-T	Ia	NCT03672305	Not yet
Advanced HCC	2015	13	China	Anti-GPC3 CAR-T	I	NCT02395250	Completed
Malignancies	2018	73	China	Autologous CAR-T/TCR	I/II	NCT03638206	Yes
PC	2017	18	US	huCART-meso cells	I	NCT03323944	Yes
HCC	2019	14	US	GPC3-CAR-T + Cytoxan + Fludarabine	I	NCT02905188	Yes
Malignant solid tumor	2019	30	China	Nectin 4/FAP CAR-T	I	NCT03932565	Yes
PC	2018	10	China	CAR-T-meso cells	N/A	NCT03638193	Yes
PC CEA + liver metastases	2019	6	US	CAR2Anti-CEA CAR-T	Ib	NCT03818165	Yes
HCC/Metastatic colorectal and PC	2016	20	China	CAR-T	I/II	NCT02959151	Unknown
EPCAM + cancer	2017	60	China	CAR-T	I/II	NCT03013712	Yes
HCC	2017	18	China	ET1402L1-CAR-T	I	NCT03888859	Yes
Metastatic CRC	2018	20	China	EGFR/CAR-T	I	NCT03542799	Not yet
Advanced malignancies	2015	20	China	Anti-CD133 CAR-T	I	NCT02541370	Unknown
**Tumor-Infiltrating Lymphocyte Therapies**							
Metastatic cancer	2010	332	US	TIL + Aldesleukin + Cyclophosphamide + Fludarabine + Pembrolizumab	II	NCT01174121	Yes
Colorectal cancer	2019	20	China	Anti-PD-1 activated TIL + Xelox	I/II	NCT03904537	Yes
Advanced refractory solid tumor	2017	40	China	Neoantigen Reactive T Cells (NRTs) + PD-1	I/II	NCT03171220	Yes
Advanced solid cancer	2019	240	US	TIL + Cyclophosphamide + Fludarabine	II	NCT03935893	Not yet
**CIK Therapies**							
PC	2013	47	China	DC/CIK + S1	I/II	NCT01781520	Completed
PC	2018	90	China	CIK + Anti- CD3-MUC1 bispecific antibody	II	NCT03509298	Yes
CRC	2012	46	China	CIK + Folfox4	IV	NCT03084809	Completed
Colon cancer	2019	20	Iran	Autologous CIK	I/II	NCT03329664	Not yet
Metastatic CRC	2017	28	Korea	Immuncell-LC	II	NCT03220984	Enrolling by invitation
HCC	2015	55	China	Autologous CIK	I/II	NCT03124498	Unknown
Resected liver cancer	2008	200	China	CIK	III	NCT00769106	Completed
Liver transplant in HCC	2019	18	Korea	Immuncell-LC	I/II	NCT03983967	Yes
**iNKT Therapies**							
Advanced solid tumor	2017	40	China	iNKT cells + CD8^+^ T cells	I/II	NCT03093688	Yes
HCC	2017	18		Adoptive iNKT therapy	I	NCT03175679	Yes
**Bispecific T cell engager**							
PC	2015	2	US	anti-CD3 × anti-EGFR, IL-2 + GM-CSF	I/II	NCT02620865	Active, NR

Abbreviations: EGFR, epidermal growth factor receptor; iNKT, invariant natural killer-T cell; CIK, cytokine induced killer cell; GM-CSF, granulocyte macrophage-colony stimulating factor; NRT, Neoantigen Reactive T Cells; MUC-1, mucin-1; TIL, tumor infiltrating lymphocyte; GPC3, glypican-3; DC, dendritic cell; EPCAM, epithelia cell adhesion molecule; NR, not recruiting; CEA, Carcinoembryonic Antigen; CART-T, chimeric antigen receptor-T cell; TCR, T cell receptor.

**Table 2:** Clinical trial efficacy result of selected immunotherapies in GI cancer.

Cancer Type	Immunotherapy	No of Participants	Phase	Clinical Outcome	Immune Response Adverse Effect	Reference
**CAR-T**						
HCC	Anti-GPC3 CAR-T	13	I	1 PR, 3 SD, 2 PD	SAE grade 3 fever in 1 pt, 1 death	[282]
CRC	CEA CAR-T	10	I	7 SD, 2SD > 30 wk, 2 TS	No IrAE	[283]
PC	HER2 CAR-T	11	I	PFS 4.8 months, 1PR, 5 SD	Reversible toxicities, mild to moderate	[284]
Advanced metastatic cancer	CD133 CAR-T	23	I	PFS 5 months, 3PR, 14SD	Controllable toxicities	[285]
**Immune Checkpoint Inhibitor**						
Advanced GC and CRC	Regorafenib +nivolumab	50	Ib	38% ORR, 3PR in GC.44% RR(GC), 29% MSS(CRC)	Grade 3/4 toxicity in 17 pts	[286]
CRC	Atezolizumab/+ or without cobimetinib versus regorafenib	363	III	mOS 8.87 months (combination group)	SEA in 40% of pts (combination group)	[287]
**NK Cell Therapy**						
GI cancer	Allogenic NK therapy + Cetuximab	9	I	2 SD, 1 PR, I DR	No DLT	[288]
**Cancer Vaccine**						
Metastatic PC	G-VAX/CRS-207	93	II	OS arm A 6.1 months vs. arm B 3.9 months	Grade 3/4 toxicity	[289]
**BiTES**						
Refractory solid tumor	EpCAM/CD3 BiTES	65	I	18 SD, 17 BR of SD, 28 PD, 1 unconfirmed PR	DLT in 15 pts, Grade 3/4 toxicity in 95% pts	[290]
**Stroma-Targeted Therapies**						
PDAC	Pegylated IL-10 + Folfox	353	I/Ib	15.5% ORR, 10.5% CR	Grade3/4 TrAEs, Grade 1/2 neuropathy	Ongoing
Advanced metastatic solid tumor	NKTR-214	28	I	SD in 14 pts, TR in 35% of pts	Grade 3 TrAE in 21.4% of pts	[291]
Advanced metastatic solid tumor	Emactuzumab/ + paclitaxel	217	I	7% ORR in combined therapy, no anti-tumor effect	No toxicity	[292]

Abbreviations: ORR, objective response rate; OS, overall survival; PR, partial response; SD, stable disease; PD, progressive disease; CR, complete response; PFS, progression free survival; mOS, mean overall survival; Rd, dissociated response; DLT, dose limiting toxicities; SAE, serious adverse event; IrAE, immune related adverse event; TrAE, treatment related adverse effect, PDAC, pancreatic ductal adenocarcinoma; TR, tumor reduction; Pts, patients; TS, tumor shrinkage.

**Table 3:** Clinical trials of other immunotherapies in GI cancer.

Cancer Type	Start Year	No of Participants	Site of Trial	Agents	Phase	ClinicalTrials.Gov Number	Recruitment Status
**Immune Checkpoint Inhibitors/Combination Strategies**							
Advanced metastatic solid tumor	2018	48	Japan	Regorafenib + nivolumab	I/II	NCT03406871	Yes
HCC	2016	90	US	Durvalumab + tremelimumab ablative therapies (TACE, RFA, cryoablation)	II	NCT02821754	Yes
MSS CRC	2019	54	US	Copanlisib + nivolumab	I/II	NCT03711058	Yes
MSI-H/dMMR or high TMB CRC	2018	54	China	PD-1 antibody + cox inhibitor	II	NCT03638297	Yes
Stage III&IV HCC	2018	40	US	Nivolumab + sorafenib	II	NCT03439891	Yes
CRC	2018	74	US	Anti-PD-L1/TGFbetaRII fusion protein M7824	I/II	NCT03436563	Yes
Stage IV PC	2019	40	US	Nivolumab + cabiralizumab + gemcitabine	II	NCT03697564	Not yet
MSS CRC	2019	64	US	Nivolumab + relatlimab	II	NCT03642067	Yes
Advanced HCC	2019	545	AstraZeneca locations	Durvalumab + tremelimumab	II	NCT02519348	Yes
**NK-Based Therapies**							
CRC	2018	54	US	Allogenic NK + ALT803 (IL-15)	I	NCT02890758	Yes
Solid tumor	2018	30	China	NKG2D CAR-NK	I	NCT03415100	Yes
Relapse or refractory solid tumor	2016	10	China	Anti-MUC1 CAR-pNK	I/II	NCT02839954	Unknown
**Cancer Vaccines**							
Early and intermediate HCC	2017	40	Multiple locations	IMA970A + CV8102 adjuvant	I/II	NCT03203005	Yes
Refractory cancerCRC	2017	35	US	Pexa-Vec + durvalumab + tremelimumab	I/II	NCT03206073	Yes
PC	2016	26	US	LOAd703 oncolytic virus + gemcitabine + nab-paclitaxel	I/II	NCT02705196	Yes
Solid tumor	2019	23	US	VSV-IFNβ-NIS and pembrolizumab	I	NCT03647163	Yes
Colon cancer	2012	3	US	Autologous cancer vaccine TGF-β	II	NCT01505166	Terminated
Advanced malignant tumor	2018	30	China	iNeo-Vac-P01	I	NCT03662815	Yes
CRC	2005	37	Taiwan	CEA pulsed dendriticcells	I/II	NCT00154713	Unknown
**Stroma-Directed Therapies**							
MSS CRC	2018	20	Germany	CCR5 inhibitor + pembrolizumab	I	NCT03274804	Active, NR
Malignant solid tumor	2019	30	China	Nectin 4/FAP CAR-T	I	NCT03932565	Yes
Advanced PDAC	2019	30	US	Nivolumab + CCR2/CCR5 dual antagonist + G-VAX	I/II	NCT03767582	Not yet
PC	2008	12	US	G-VAX vaccine + cyclophosphamide + pembrolizumab, + anti-CSF-1R monoclonal antibody IMC-CS4	I	NCT03153410	Yes
Metastatic PC	2017	566	Multiple locations	Pegylated IL-10 + folfox	III	NCT02923921	Yes
PC	2017	140	US	Tocilizumab + gemcitabine+ nab-paclitaxel	II	NCT02767557	Yes
Metastatic PC	2017	9	US	IL-12 gene therapy	I	NCT03281382	Yes
HCC	2018	35	China	Chiauranib	1	NCT03245190	Yes
Advance cancer	2018	280	Multiple locations	Anti-IL-8 + Nivolumab	I/IIa	NCT03400332	Yes

Abbreviations: HCC, hepatocellular carcinoma; TACE, transarterial chemoembolization; RFA, radiofrequency ablation; MSS, microsatellite stable; MSI-H, microsatellite instability; dMMR, mismatched repair deficiency; TMB, tumor mutation burden; PD-1, programmed cell death-1; COX, cyclo-oxygenase; CRC, colorectal cancer; PC, pancreatic cancer; NK, natural killer; CAR, chimeric antigen receptor; CAR-NK, chimeric antigen receptor –natural killer; MUC-1, mucin 1; CEA, Carcinoembryonic Antigen; FAP, fibroblast activation protein; CCR2, C-C chemokine receptor type 2; C-C chemokine receptor type 5; CSF-1R,colony stimulating factor-1 receptor; IL—interleukin; TGF-β, transforming growth factor; CAR-T; chimeric antigen receptor-T cell; VSV-IFNβ-NIS, Oncolytic VSV engineered to express interferon-beta (IFNβ) and the sodium iodide symporter (NIS); NKG2D, Natural killer group 2 member D.
